# Jagged-1 signaling suppresses the IL-6 and TGF-β treatment-induced Th17 cell differentiation via the reduction of RORγt/IL-17A/IL-17F/IL-23a/IL-12rb1

**DOI:** 10.1038/srep08234

**Published:** 2015-02-04

**Authors:** Yuan Wang, Feiyue Xing, Siqi Ye, Jia Xiao, Jingfang Di, Shan Zeng, Jing Liu

**Affiliations:** 1 Institute of Tissue Transplantation and Immunology, Department of Immunobiology, Jinan University, Guangzhou 510632, China; 2 Key Laboratory of Functional Protein Research of Guangdong Higher Education Institutes, Jinan University, Guangzhou 510632, China; 3 Department of Stomatology, Jinan University, Guangzhou 510632, China

## Abstract

Jagged-1 signaling has recently been reported to be involved in the Th17 cell differentiation. However, little is known about its mechanisms. Soluble Jagged-1 was used to activate the Jagged-1–Notch signaling to interfere with the IL-6 and TGF-β-induced Th17 cell skewing. Genes relevant to the autoimmunity or inflammation were screened for the first time in this system by qPCR array for the differential expressions. The 18 genes out of 84, including *Clec7a, Il12b, Il12rb1, Il12rb2, Csf3, Il15, Il17a, Il17f, Il17rc, Il17rd, Il17re, Il23a, Myd88, Socs1, Stat4, Stat5a, Sykb* and *Tbx21*, were downregulated, but only *Cxcl2*, *Cxcl12* and *Mmp3* were upregulated. The expressions of the genes, *Rorγt*, *Il17a*, *Il17f*, *Il12rb1* and *Il23a*, induced by simultaneous IL-6 and TGF-β treatment were significantly suppressed by Jagged-1, followed by the reduction of RORγt, IL-17A, and IL-17F. Consistent with the attenuation of RORγt, and the reduced production and secretion of IL-17A and IL-17F in the cell supernatant and the *in situ* stained cells, the number of CD4^+^IL-17^+^ cells was also diminished. It is concluded that the Jagged-1–Notch signaling can suppress the IL-6 and TGF-β treatment-induced Th17 cell skewing through the attenuation of RORγt and, hence by, the down-regulation of IL-17A, IL-17F, IL-23a, and IL-12rb1.

Recently, the Th1/Th2 paradigm has been expanded, following the discovery of a third subset of the effector Th cells, called Th17 cells^1^,^2^,^3^ and characterized by the production of IL-17A, IL-17F, and IL-22 as the signature cytokines. In the presence of TGF-β alone, naive T cells express Foxp3 that induces the regulatory T cells^4^. A relevant finding is that IL-6 is a potent inhibitor of the TGF-β-driven induction of Foxp3^+^ regulatory T cells^5^. IL-6 not only inhibits the generation of these cells, but also together with TGF-β, guides the naive CD4^+^ T cells to produce IL-17. Th17 cells express a unique transcription factor, RORγt^6^, which induces the transcription of the *Il-17* gene in naive helper T cells to promote the development of IL-17-producing cells in the presence of TGF-β and IL-6^7^. RORγt activation also induces the expression of IL-23R, indicating that IL-23 acts on T cells that are already committed to the Th17 lineage^8^. It is well known that the Th17 cells play a key part in the pathogenesis of the autoimmune and inflammatory diseases as well as the tumors. Thus, it is of great significance to reveal the mechanisms that regulate the Th17 cell differentiation.

Notch signaling has been proven to decide the fates of immune cells. In mammals, there are four Notch receptors, including Notch-1, Notch-2, Notch-3 and Notch-4, and five Notch ligands, including Jagged-1, Jagged-2, delta-1 (Dll1), delta-3 (Dll3) and delta-4 (Dll4). Jagged-1 is taken as the example. While Jagged-1 interacts with the Notch-1 or Notch-2, successive cleavages are triggered in the transmembrane region of Notch receptors by the disintegrin and metalloproteinase. The endocytosis of the transmembrane fragment of the Notch contributes to the further cleavage by γ-secretase, leading to the release of the Notch intracellular domain and the final activation of the Notch signaling pathway.

Radtke *et al*. found that the deletion of Notch-1 resulted in the marked decrease in the size of thymus that lacked T cells and contained the excess of B cells^9^,^10^. Notch-1 inactivation causes a complete block in T lineage development, indicating that the other Notch family members cannot compensate for the loss of Notch-1 *in vivo*^10^. Notch signaling not only regulates the T/B cell lineage commitment, but also the differentiation and function of the peripheral T cells^11^,^12^. Different Notch ligands play various roles in the differentiation of the naive CD4^+^ T cells. The antigenic stimulation of the naive CD4^+^ T cells in the context of antigen-presenting cells engineered to express Dll1 leads to the secretion of Th1 cytokines, such as IFN-γ^13^, whereas the Jagged-1 promotes the Th2 cytokine production, such as IL-4^14^. Mukherjee *et al*. found that the dendritic cells (DCs) were activated by TLR-specific signals, and Dll4 up-regulated the RORγt expression in T cells; both *rorγt* and *il17* gene promoters were the direct transcriptional notch targets that further enhance the differentiation of Th17 cell populations, while anti-Dll4Ab significantly inhibited the differentiation^15^. Ito *et al*. showed that the TLR9-deficient mice challenged with a mycobacterium antigen displayed an altered Th17 cytokine profile, the decreased accumulation of granuloma-associated myeloid DCs, and profoundly reduced Dll4 expression, suggesting that the Dll4 plays an important role in promoting the Th17 activity during mycobacterium challenge^16^. Notch-3 antibody could down-regulate the expression of IL-17 in experimental autoimmune encephalomyelitis. These data suggest that the Dll-Notch signaling may be involved in the differentiation of Th17 cells^17^. Our previous study showed that the Jagged-1–Hes-1 signaling could reduce the production of IL-17 in CD4^+^ T cells, which was reversed by Hes-1-targeting siRNA^18^. However, it is still unclear how genes relevant to autoimmunity or inflammation are altered in the Jagged-1-treated cells. Therefore, in the current study, qPCR array was used for the first time to explore the genes that may play an important part in the Jagged-1-mediated Th17 cell differentiation from different angles.

## Results

### Jagged-1–Notch signaling inhibits the differentiation of the CD4^+^ T cells into Th17 cells

**Figure 1:**
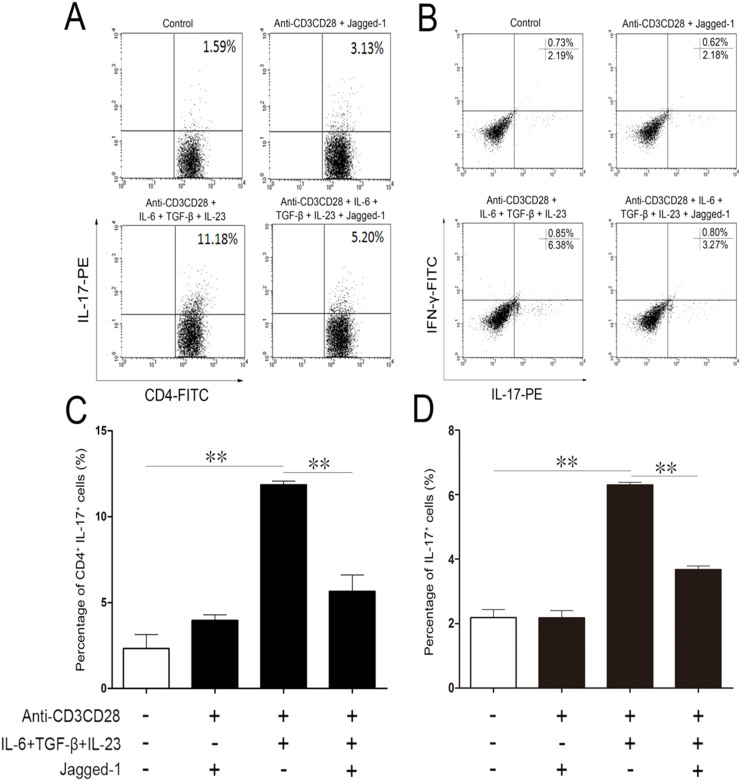
The inhibition of CD4^+^ T cells towards Th17 cell differentiation by Jagged-1 signaling. The isolated CD4^+^ T cells were treated by Jagged-1 and quantitatively analyzed by the flow cytometry, 72 h after the treatment. (A) The levels of intracellular IL-17 in the treated CD4^+^ T cells. (B) Intracellular IL-17 and IFN-γ production in the treated CD4^+^ T cells. (C) Percentage of CD4^+^IL-17^+^ T cells. (D) Proportion of IL-17^+^ cells in the lower right quadrant in the FACS plots. Three independent experiments were repeated. **p < 0.01, *vs.* the corresponding control.

Flow cytometry was performed to analyze the phenotypes of CD4^+^ T cells treated with Jagged-1. As shown in Fig. 1, compared with the anti-CD3/CD28 group, the group, treated simultaneously by IL-6 and TGF-β, showed the increased percentage of CD4^+^IL-17^+^ T cells. This augment can be abolished by Jagged-1. However, the percentage of IFN-γ^+^IL-17^+^ T cells was unchanged in each group. The results indicate that the activation of the Jagged-1 signaling can inhibit the polarization of Th17 cells without the enhancement of Th1-like cytokine.

### Jagged-1–Notch signaling elicits the differential expression in genes in the CD4^+^ T cells

**Figure 2:**
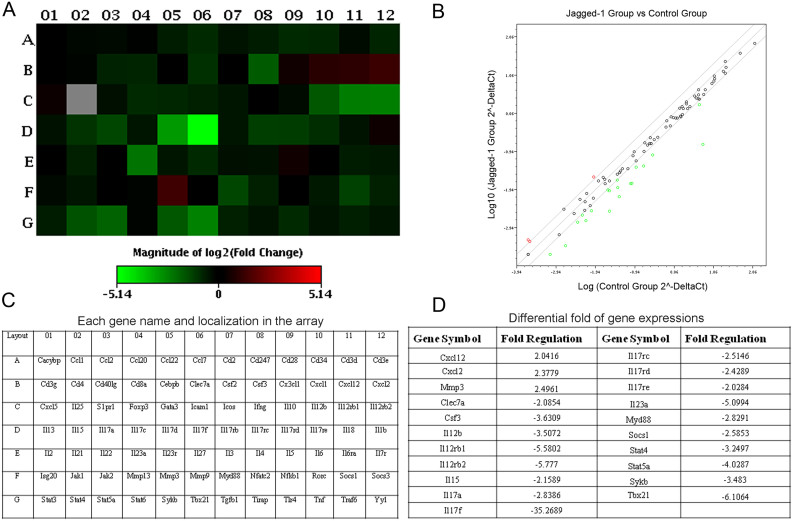
Differential expressions of the genes relevant to the Th17 cell differentiation by Jagged-1 signaling. (A) The heat map provides a graphical representation of expression fold between the Jagged-1-treated CD4^+^ T cells (Jagged-1 group) and the untreated CD4^+^ T cells (control group). Red indicates the upregulated genes and green, the downregulated genes. (B) The both groups are overlaid onto the qPCR Array plate layout with genes' name and their location. (C) The relative expressional levels for each gene in the Jagged-1 group and the control group are plotted against each other in the scatter plot. Red shows the upregulated genes and green, the downregulated genes. (D) Up/downregulated genes in CD4^+^ T cells from the Jagged-1 group are compared with the control group. Fold-change values greater than two hint the positive regulation, and fold-change values less than two, the negative regulation. Two independent experiments were repeated.

Among the assessed 84 genes, 21 genes were markedly changed with the changes greater than 2.0 fold, including 3 upregulated genes, ie. chemokine (C-X-C motif) ligand 12 (*cxcl12*), chemokine (C-X-C motif) ligand 2 (*cxcl2*) and matrix metallopeptidase 3 (*mmp3*); and 18 downregulated genes, ie. c-type lectin domain family 7 member a (*clec7a*), colony stimulating factor 3 (*csf3*), interleukin 12b (*il12b*), interleukin12 receptor beta1 (*il12rb1*), interleukin 12 receptor beta 2 (*il12rb2*), interleukin 15 (*il15*), interleukin 17a (*il17a*), interleukin 17d (*il17d*), interleukin 17f (*il17f*), interleukin 17 receptor C (*il17rc*), interleukin 17 receptor D (*il17rd*), interleukin 17 receptor E (*il17re*), interleukin 23 alpha (*il23a*), myeloid differentiation primary response gene 88 (*myd88*), suppressor of cytokine signaling 1 (*socs1*), signal transducer and activator of transcription 4 (*stat4*), signal transducer and activator of transcription 5a (*stat5a*), spleen tyrosine kinase (*sykb*) and T-box 21 (*tbx21*). Especially, there was 3-fold downregulation in the expression of IL17a, but over 35-fold in that of IL17f. The differential gene expressions in the CD4^+^ T cells treated with Jagged-1 were showed in HeatMap and ScatterPlot, respectively (Fig. 2A–B). Their names and the significant differences in the gene expression profiles in CD4^+^ T cells between Jagged-1 and control groups were presented in Fig. 2C–D. The data indicate that the activation of Jagged-1 signaling results in the downregulation of some genes (*clec7a, il12b*, *il12rb2* and *il15*) to promote the proliferation of T cells or NK cells; and some genes (*tbx21, stat4, il12b, il12rb1, il12rb2, il17a, il17f, il17rc* and *il23a*) to influence the polarization and stability of Th1 cells or Th17 cells, and the other genes (*il17rd* and *il17re*) to activate the MAPK signaling pathway. Additionally, we also noted that *cxcl12* (with chemotactic influence on T cells and monocytes), *cxcl2* (with chemotactic impact on neutrophil) and *mmp3* (with hydrolysis of the collagen) were upregulated, the significance of which is unclear.

### The reduction in the expressions of *il-17a*, *il-17f*, *il-12rb1* and *il-23a* by Jagged-1–Notch signaling

**Figure 3:**
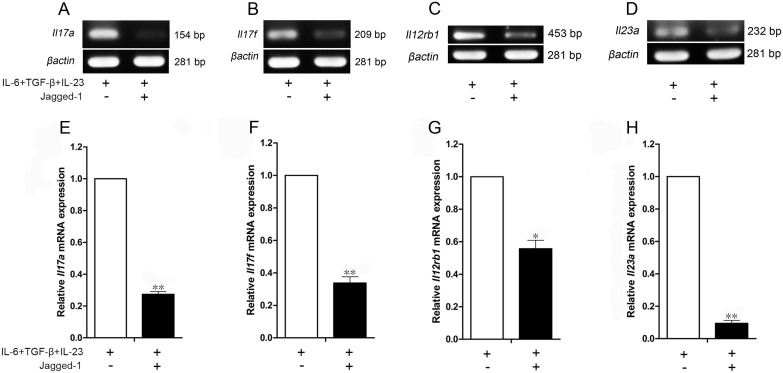
The decreased expressions of *Il17a*, *Il17f*, *Il12rb1* and *Il23a* genes in the Jagged-1-treated CD4^+^ T cells. (A–D) The expressions of *Il17a*, *Il17f*, *Il12rb1* and *Il23a* mRNA were evaluated by RT-PCR, and the PCR products were separated by electrophoresis on the 1.5% agarose gel. (E–H) the relative quantification of *Il17a*, *Il17f*, *Il12rb1* and *Il23a* mRNA expressions was performed using the 2^−ΔΔCt^ method by qPCR. The results are representative of the three independent experiments. *p < 0.05, **p < 0.01 *vs.* the corresponding control.

The Jagged-1–Notch-1 signaling induced changes in *il-17a*, *il-17f*, *il-12rb1* and *il-23a* were further confirmed by RT-PCR (Fig. 3A–D) and qPCR (Fig. 3E–H). As shown in Fig. 3, compared with the group treated with IL-6/TGF-β/IL-23a in the presence of anti-CD3/CD28, the activation of Jagged-1–Notch signaling indeed led to the decrease of *il-17a*, *il-17f*, *il-12rb1* and *il-23a* levels in the CD4^+^ T cells, consistent with the alterations observed by qPCR array. However, there was no difference between IL-17A and IL-17F, different from the qPCR array data. These results show that the Jagged-1 can inhibit the Th17 cell differentiation, maturation and stability.

### Jagged-1–Notch-1 signaling inhibits the specific cytokine production in Th17 cells via RORγt

**Figure 4:**
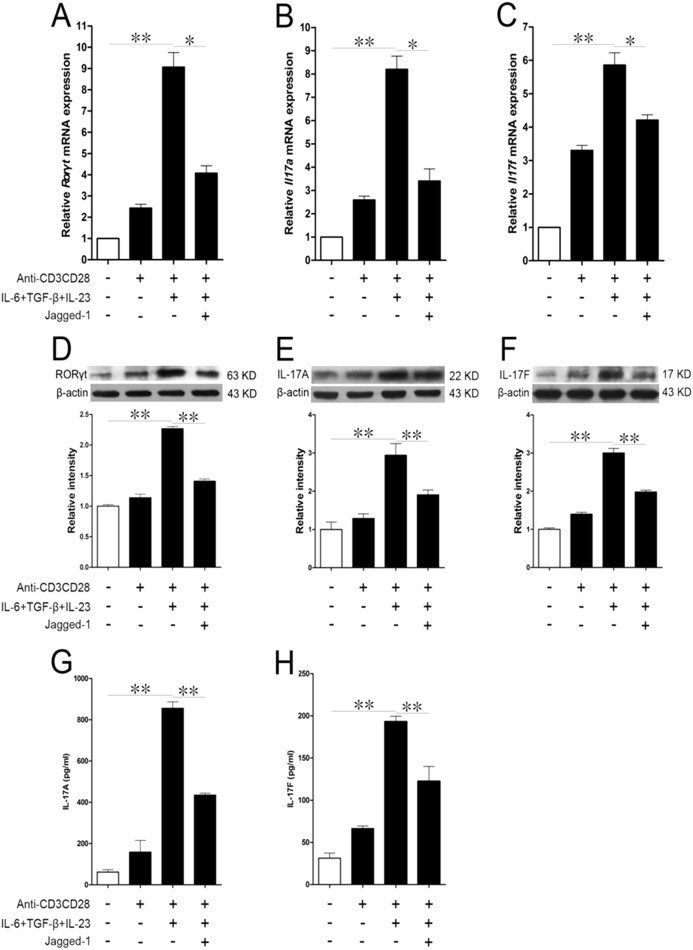
The downregulation of IL-17A, IL-17F and the RORγt expressions in the Jagged-1-treated CD4^+^ T cells. The isolated CD4^+^ T cells were treated with Jagged-1 for 72 h. (A–C) The relative quantification of *Il-17a*, *Il-17f* and *Rorγt* mRNA expressions was detected using 2^−ΔΔCt^ method by qPCR. (D–F) The levels of IL-17A, IL-17F and RORγt proteins were determined by Western Blot. (G and H) The levels of IL-17A and IL-17F secreted by the treated CD4^+^ T cells were measured by ELISA. The results are representative of the three independent experiments. *p < 0.05, **p < 0.01.

IL-17A and IL-17F were considered as functional executants of Th17 cells. qPCR and Western Blot were first used to evaluate the relationship among RORγt, IL-17A and IL-17F. Compared with the anti-CD3/CD28 group, both IL-6 and TGF-β could obviously increase the expressions of RORγt, IL-17A and IL-17F mRNAs, but Jagged-1 could decrease their expressions in the CD4^+^ T cells induced by simultaneous IL-6 and TGF-β treatment (Fig. 4A–C). Following the alterations in RORγt, IL-17A and IL-17F mRNAs, RORγt, IL-17A and IL-17F protein levels were also decreased. Both IL-6 and TGF-β upregulated the expressions of RORγt, IL-17A and IL-17F, whereas this augment could be abrogated by Jagged-1 (Fig. 4D–F). Furthermore, the secretion of IL-17A and IL-17F in the treated cells was tested by ELISA. Compared with the anti-CD3/CD28 group, simultaneous IL-6 and TGF-β treatment boosted the cells to secrete IL-17A and IL-17F, which could be reversed by Jagged-1 (Fig. 4G–H). These findings further support that the Jagged-1–Notch-1 signaling inhibits the production of IL-17A and IL-17F in the CD4^+^ T cells via RORγt. The qPCR array data showed that the level of IL-17F in the treated cells was lower than that of IL-17A. It seems that IL-17F might play a more important role in Th17 cell polarization, but other results demonstrate that there is no significant difference between the both.

### Jagged-1–Notch-1 signaling inhibits the *in situ* expressions of RORγt, IL-17A and IL-17F induced by simultaneous IL-6 and TGF-β treatment

**Figure 5:**
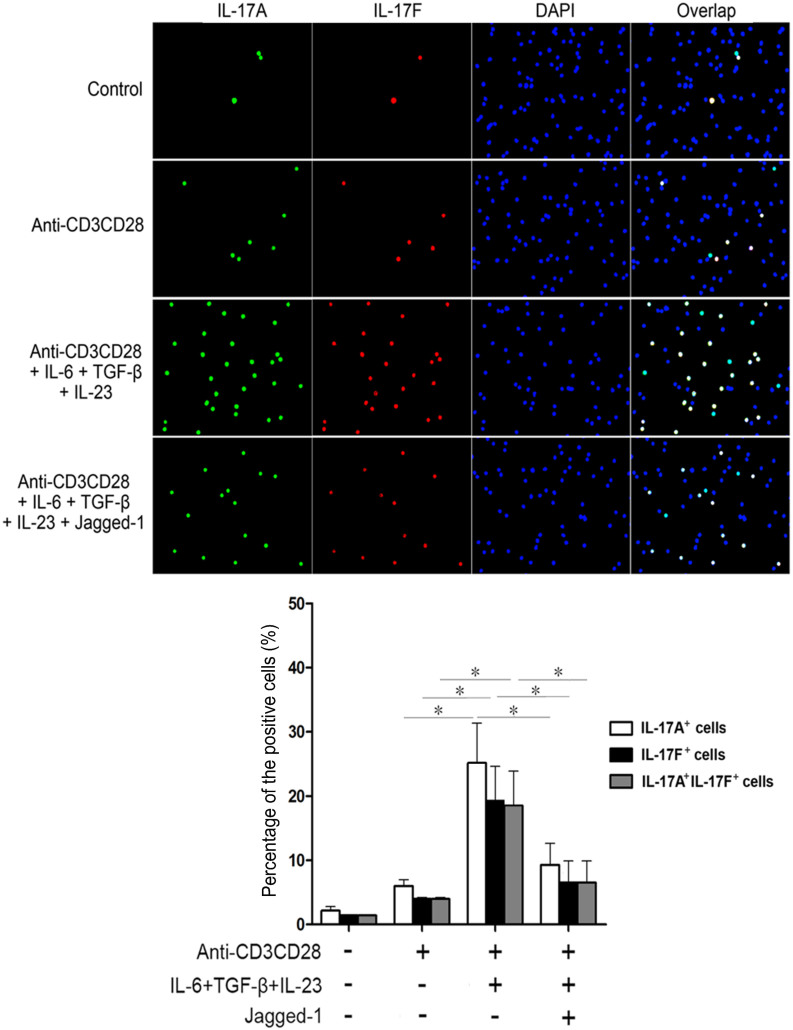
The *in situ* localization and distribution of IL-17A and IL-17F in the CD4^+^ T cells treated with Jagged-1. The isolated CD4^+^ T cells were treated with Jagged-1 for 72 h. The cells were fixed, permeablized and stained with monoclonal antibodies specific for IL-17A (green) and IL-17F (red). The nuclei were stained with DAPI (blue). All the cells were observed at magnification ×200. Three independent experiments were repeated. *p < 0.05, *vs.* the corresponding control.

**Figure 6:**
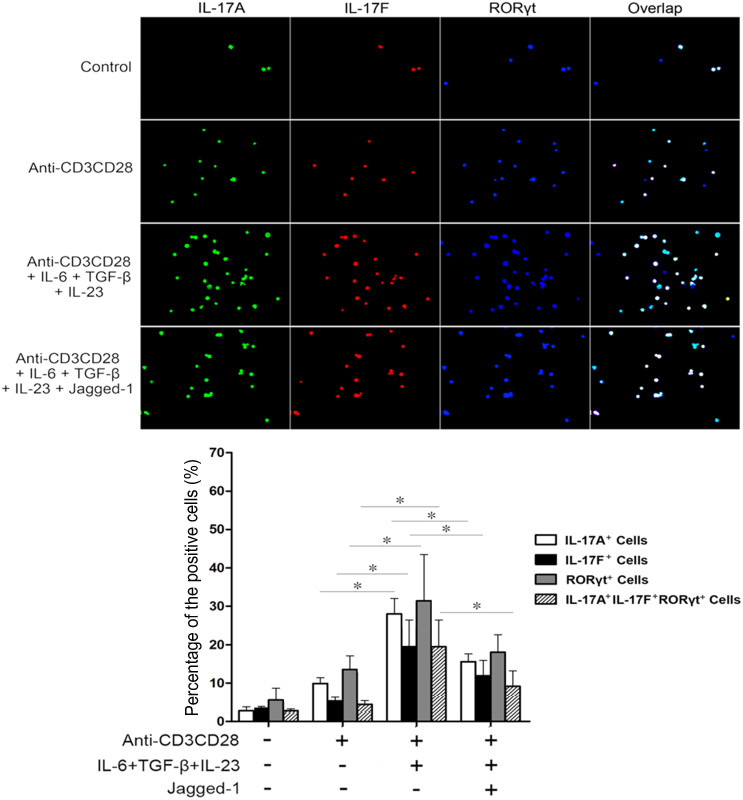
The *in situ* expressional relationship of both IL-17A and IL-17F to RORγt in the CD4^+^ T cells treated with Jagged-1. The isolated CD4^+^ T cells were treated with Jagged-1 for 72 h. The cells were fixed, permeablized and stained with monoclonal antibodies specific for IL-17A (green), IL-17F (red) and RORγt (blue). All the cells were observed at magnification ×200. Three independent experiments were repeated. *p < 0.05, *vs.* the corresponding control.

As shown in Fig. 5–6, CD4^+^ T cells were treated with IL-6, TGF-β and Jagged-1 for 72 h to find out the effects of Jagged-1–Notch-1 signaling on the *in situ* expressions of RORγt, IL-17A and IL-17F in the CD4^+^ T cells. Compared with the anti-CD3/CD28 group, the group, treated simultaneously by IL-6 and TGF-β, showed the enhanced *in situ* expressions of RORγt, IL-17A, and IL-17F. The *in situ* expressions of RORγt, IL-17A and IL-17F in the CD4^+^ T cells induced by simultaneous IL-6 and TGF-β treatment were obviously decreased after the treatment by Jagged-1. Moreover, the changes in IL-17A and IL-17F levels were consistent with the RORγt expression. The data further support that the activation of Jagged-1–Notch-1 signaling pathway may inhibit the differentiation of CD4^+^ T cells towards Th17 cells induced by simultaneous IL-6 and TGF-β treatment via the reduction of RORγt/IL-17A/IL-17F/IL-23a/IL-12rb1.

## Discussion

Naive CD4^+^ T cells express Notch-1 and Notch-2 receptor mRNAs, while they do not express Notch-3 and Notch-4 mRNAs^14^. T cells express Notch ligands Jagged-1^24^, Jagged-2^25^, and Delta-1^26^. DCs treated with lipopolysacride can express Notch ligands Jagged-1 and Jagged-2 as well as Delta-4^14^,^24^,^25^. Jagged-1 was originally isolated as a mammalian ligand that activates the Notch-1 signaling. The activated domain has been mapped to a N-terminal extracellular region of Jagged-1, as a specific peptide from the Delta/Serrate/Lag2 (DSL)-domain of Jagged-1 that can mediate the activation of Notch signaling^27^.

There are debates about the suppression of T cell activation or the enhancement of T cell function by Notch signaling in murine and human systems^28^. Notch ligands have been shown to differently influence the T cell differentiation^29^,^30^,^31^. Yasutomo *et al*. first demonstrated that Dll1 could promote the skewing of naive CD4^+^ T cells toward Th1^29^. Subsequently, Amsen *et al*. found that Jagged-1 could induce naïve CD4^+^ T cells to differentiate into Th2 lineage by increasing the expression of IL-4 in naïve CD4^+^ T cells^14^. Other investigators showed that Jagged-1 directly activated a Jagged-1–Notch signaling pathway, inducing naïve peripheral T cells to differentiate into regulatory T cells^30^,^31^. Furthermore, Ito *et al*. found that Dll4 would improve the secretion of Th17 cytokines, such as IL-17A and IL-17F *in vitro* and *in vivo*. Dll4 influences the generation of IL-17-producing T cells in the presence of both IL-6 and TGF-β^16^. Our previous data indicate that the Jagged-1-Hes-1 signaling can suppress the skewing of CD4^+^ T cells toward Th17 cells by means of siRNA to knockdown *hes-1* gene, but the genes that function in the Th17 cell skewing remain poorly understood^18^. In this study, we observed the alterations in 84 genes possibly related to the differentiation of Th17 cells for the first time to find out the genes that play a prominent part in the differentiation induced by Jagged-1. The novel findings demonstrate that *cxcl12, cxcl2 and mmp3* gene expressions are increased, but *clec7a, csf3, il12b, il12rb1, il12rb2, il15, il17a, il17d, il17f, il17rc, il17rd, il17re, il23a, myd88, socs1, stat4, stat5a, sykb and tbx21* expressions are decreased. Some of the downregulated genes, such as *il12b, il12rb1, il12rb2, stat4 and tbx21*, might be related to Th1 cell polarization, but others, mainly including *il17a, il17d, il17f, il17rc, il17rd, il17re and il23a*, might be related to Th17 cell skewing. Furthermore, *il17a*, *il17f*, *il12rb1* and *il23a* were selected for further confirming their relationship to Jagged-1 signaling. They were indeed reduced in the treated cells, which is consistent with the attenuation of CD4^+^IL17^+^ T cells. The downregulation of IL17a, IL17f, IL17rc, IL17rd and IL17re contributes to the inhibition of Th17 cell skewing by Jagged-1. Are they correlated to RORγt change? The reductions in both IL17A and IL17F are attributed to the decreased expression of RORγt by Jagged-1, and that Jagged-1 signaling is responsible for the reduction in IL12rb1, which may avail the inhibition of naïve CD4^+^ T cell skewing toward Th1. In addition, the reduction in IL23a by Jagged-1 may preclude the maturation of Th17, contributing to the decreased number of CD4^+^IL17^+^ T cells by Jagged-1 signaling, too. Undoubtedly, these findings provide a novel insight into the mechanisms of Th17 cell differentiation. Of course, it merits to exploring further the roles that Cxcl12, Cxcl2 and Mmp3 play in the Jagged-1-inhibited Th17 cell skewing.

IL-23 is a member of IL-12 family of heterodimeric cytokines. It is composed of two subunits: IL-12p40, which is common to IL-12, and IL-23-specific p19 subunit^32^. The reported relationships of IL-12 and Th1 cells with autoimmunity can be explained by the requirement for IL-23^33^. Many of the proinflammatory functions of IL-23 seem to be related to Th17 cell subset^34^. The stimulation of IL-23 maintains the IL-17 production by Th17 cells^35^, that enhances the Th17 cell differentiation^32^, or promotes the survival of Th17 cells^36^. In the presence of proinflammatory cytokines particularly, TGF-β and IL-6 can trigger the Th17 differentiation through Th17-specific transcription factor RORγt, which leads to the production of IL-17A and IL-17F^3^,^5^,^37^. IL-23R is up-regulated on the membrane of the naïve CD4^+^ T cells after activation in the presence of IL-6^6^. IL-23R is induced in the developing Th17 cells, and IL-12Rb2 is produced in the developing Th1 cells to pair with the constitutively expressed IL-12Rb1 chain to combine with IL-23 and IL-12, respectively. IL-12Rb1 chain binds a common subunit of IL-23 and IL-12 heterodimers (IL-12p40 or IL-12b) to pair with IL-23p19 (IL-23a) or IL-12p35 (IL-12a), respectively^32^,^38^,^39^. In our study, the expressions of *Il17a, Il17f, Il12rb1 and Il23a* were also significantly inhibited by Jagged-1, which was directly related to the observed Th17 phenotype, suggesting that the IL-23 signaling pathway plays a role in the development of Th17 cells. Some reports suggest that the activation of the IL-23 or IL-12 receptor induces late events in the downstream of early Th17 or Th1 lineage commitment^1^,^37^. Whereas the IL-12 receptor potently activates Stat4 in Th1 cells, the IL-23 receptor predominantly activates Stat3, but also recruits some Stat4^32^,^40^,^41^. Stat3 is required for programming TGF-β1 and IL-6, and the IL-23-stimulated IL-17-secreting phenotype. Moreover, the retroviral transduction of the constitutively active Stat3 into the differentiating T cells enhances IL-17 production, suggesting that the Stat3 may directly regulate the forming of Th17 cells^40^. Moreover, the IFN-γ- and IL-17-producing T cells were typically found in association with the IFN-γ-producing T cells in the context of both autoimmune inflammation and infection. These data suggest that there might be a parallel between Th1 and Th17 developmental programs. Our results show that the Jagged-1 does not influence the percentage of IFN-γ-producing CD4^+^ T cells, but significantly decreases the number of IL-17-producing CD4^+^ T cells. The critical cytokines and their receptors to promote Th1 cell skewing, including IL12b, IL12rb1 and IL12rb2, are reduced, and the crucial transcriptional factors to facilitate Th1 cell skewing, including Stat4 and T-box 21 are also attenuated, further supporting our findings.

Although several up to date reports implicate the impact of Notch signaling on the Th17 differentiation, there still exist the opposite effects. Elyaman *et al.* reported that Jagged-1 could protect from the Experimental Autoimmune Encephalomyelitis (EAE), but Dll1 exacerbated its development. On the contrary, the administration of anti-Jagged-1 Ab exacerbated the EAE, while anti-Dll1 Ab reduced its severity. These data suggest that Jagged-1 may inhibit Th17 cell deviation or function^42^. In our study, Jagged-1 could reduce the number of CD4^+^IL-17^+^ T cells with the reduction of IL-17A and IL-17F, and did not influence the expression of IFN-γ, indicating that it may suppress the skewing of CD4^+^ T cells toward Th17 nor Th1 cells. Keerthivasan *et al*. obtained the opposite evidence that if Notch signaling was blocked; the production of Th17-associated cytokines was diminished, suggesting that the Notch signaling is required for the Th17 differentiation^43^. Of note, Bailis' team found that Notch did not direct the Th cell differentiation, but instead concurrently regulated the Th1, Th2, and Th17 cell genetic programs mainly by the addition of a gamma secretase inhibitor^44^. Through comparison and analysis for the associated reports, we consider that this contradiction or difference might be the result from the differentially polarizing conditions, dissimilarly inducing protocols, different host cells (or models) and evaluating standards.

Taken together, 84 genes relevant to the autoimmunity and inflammation were screened in this study for the first time to explore the potential roles of these genes in the Jagged-1-inhibited Th17 cell differentiation. The novel findings indicate that the activation of Jagged-1 signaling leads to the downregulation of *clec7a, il12b, il12rb1, il12rb2, csf3, il15, il17a, il17f, il17rc, il17rd, il17re, il23a, myd88, socs1, stat4, stat5a, sykb and tbx21*, and the upregulation of *cxcl12*, *cxcl2* and *mmp3* under Th17 cell polarization, and that the Jagged-1 signaling suppresses the simultaneous IL-6 and TGF-β treatment-induced Th17 cell differentiation via the reduction of RORγt/IL-17A/IL-17F/IL-23a/IL-12rb1. The results provide a new insight into Th17 cell differentiation and a potential strategy to inhibit its deviation. Additionally, our results also suggest that the correlation between the screened Il12rb1/Clec7a/Socs1 and RORγt deserves to be further explored in the Jagged-1-inhibited Th17 polarization.

## Methods

### Mice

BALB/c mice were purchased from the Guangzhou Medical Animal Center (Guangzhou, China). Eight week-old animals were used. All animals were bred and maintained under the specific pathogen-free condition. All animal handling and experiment procedures were approved by the Animal Care and Use Committee of Guangzhou Medical Animal Center. All experiments below were performed in accordance with relevant guidelines and regulations.

### Immunomagnetic bead isolation

Peripheral lymph nodes in mice were triturated mechanically and filtered by using the 200 meshes of stainless wire net for the isolation of lymphocytes. Then, the cells were harvested, washed twice with PBS, and re-suspended in Roswell Park Memorial Institute 1640 complete culture medium containing 10% (v/v) fetal bovine serum (Gibco BRL, Gaithersburg, MD, USA) at the concentration of 1 × 10^6^ cells/ml.

CD4^+^ T cells were segregated from the above separated lymphocytes using the mouse naïve CD4^+^ T cell isolation kit (EasySep Negative Selection Kit, StemCell, Vancouver, BC, CA). The lymphocytes were suspended at a concentration of 1 × 10^8^ cells/ml with medium plus 5% normal rat serum in a polystyrene tube. According to the manufacturer's instructions, CD4^+^ T Cell Enrichment Cocktail, Biotin Selection Cocktail and Magnetic Nano particles were added into the tube, respectively. The CD4^+^ T cells were enriched in suspension and the unwanted cells were discarded through magnetic labeling. Then, the purity of the separated CD4^+^ T cell population was tested under a flow cytometer, reaching 98%. The collected CD4^+^ T cells were cultured at 37°C in 5% CO_2_ in the complete medium.

### Flow cytometry

The cells were treated with or without 1 μg/ml Jagged-1 (R&D Systems, Minneapolis, MN, USA) for 72 h in the presence of anti-CD3/CD28 beads at a bead-to-cell ratio of 1:1 (Invitrogen, Carlsbad, CA, USA), 20 ng/ml IL-6, 10 ng/ml TGF-β, 20 ng/ml IL-23 (PeproTech, Rocky Hill, NJ, USA), 10 μg/ml anti-IFN-γ and 10 μg/ml anti-IL-4 (eBioscience, San Diego, CA, USA). The additional cells were treated with the equal volume of phosphate buffered saline (PBS) as a control. Six hours before the end of the treatment, the cells were stimulated with 50 ng/ml phorbol-12-myristate-13-acetate (PMA) (Sigma-Aldrich, St. Louis, MO, USA) plus 1 μg/ml of ionomycin (Alexis, Lausen, Switzerland) for the below experiments. Meanwhile, 10 μg/ml of GolgiStop (Becton Dickinson, Franklin Lakes, NJ, USA) was added to the cells. The cells were washed with PBS and stained with FITC-conjugated anti-mouse CD4 (0.125 μg per million cells) at 37°C for 20 min. The cells were washed, fixed, permeabilized with Fixation/Permeabilization Buffer and intracellular-stained with PE-conjugated anti-mouse IL-17 (0.05 μg per million cells) and FITC-conjugated anti-mouse IFN-γ (0.25 μg per million cells) (eBioscience, San Diego, CA, USA) for 30 min at 4°C, and analyzed with a flow cytometer (FACSCalibur, Becton Dickinson, Mountain View, CA, USA).

### Quantitative PCR array

The isolated CD4^+^ T cells were treated with or without 1 μg/ml Jagged-1 for 72 h. Total RNA was extracted using the Trizol RNA Extraction Reagent (Invitrogen, Carlsbad, CA, USA) according to the manufacturer's instructions. RNA samples were tested for the concentration and purity by Nanodrop 2000 (ThermoFisher Scientific). One microgram of the total RNA was reverse-transcribed to obtain cDNA with a RT^2^ First Strand Kit (QIAGEN GmbH, Hildden, Germany), and applied to PCR array plates. Then, the 84 genes examined using RT^2^ Profiler PCR Array Mouse Th17 for Autoimmunity and Inflammation (PAMM-073A, Qiagen, Germany) are listed in Table 1 beside 12 genes as genomic DNA contamination control, reverse transcription control and internal control, respectively. 2700 μl of qPCR mixture contained 102 μl of cDNA, 1350 μl of 2 × RT^2^ SYBR Green Mastermix, and 1248 μl of RNase free water. qPCR mixture was added to the array at 25 μl/well. The qPCR reaction conditions were as follows: 95°C for 10 min, 95°C for 15 s, 55°C for 40 s, 72°C for 30 s, and 40 cycles in total. The qPCR was performed on a Bio-Rad/MJ Research Chromo4 (BIO-RAD, Berkeley, CA, USA), and each sample with and without Jagged-1 treatment was repeatedly tested using the array for average of CT values. The 2^−ΔΔCT^ method was performed for the relative quantification of the mRNA expression using the web-based software for the cataloged and custom arrays (http://pcrdataanalysis.sabiosciences.com/pcr/arrayanalysis.php) (Qiagen, Germany). The gene expressions of the Jagged-1-treated group with changes greater than 2.0 fold were obtained, compared with the control group.

### RT-PCR and real-time quantitative PCR

To further confirm the changes of the related genes in the CD4^+^ T cells treated with Jagged-1, the cells were treated with or without 1 μg/ml Jagged-1 in the presence of anti-CD3/CD28, IL-6, TGF-β and IL-23 for 72 h, and the total RNA was extracted using a Trizol RNA Extraction Reagent (Invitrogen, Carlsbad, CA, USA). One microgram of the total RNA was used in each reaction primed with oligo-dT to obtain cDNA. Then, 1 μl of the synthesized cDNA was used as a template for RT-PCR or real-time quantitative PCR. The primers were as follows: *Il-17a*^19^ sense 5′-TCCCTCTGTGATCTGGGAAG-3′, *Il-17a* antisense 5′-CTC GAC CCT GAA AGT GAA GG-3′; *Il-17f*^6^ sense 5′-GAG GAT AAC ACT GTG AGA GTT GAC-3′, *Il-17f* antisense 5′-GAG TTC ATG GTG CTG TCT TCC-3′; *Il-23a*^20^ sense 5′-TGG CAT CGA GAA ACT GTG AGA-3′, *Il-23a* antisense 5′-TCA GTT CGT ATT GGT AGT CCT GTT A-3′; *Il-12rb1*^21^ sense 5′-CGG GGG TCC TGA CGC AAT ACG-3′, *Il-12rb1* antisense 5′-CTC CGG GCA TCT CGA CCA CCA G-3′; *Rorγt*^22^ sense 5′-GCT GTC AAA GTG ATC TGG AG-3′, *Rorγt* antisense 5′-GGT GGA ACT TAT GGG AAA TC-3′; *β-actin*^23^ sense 5′-AAC AGT CCG CCT AGA AGC AC-3′, *β-actin* antisense 5′-CGT TGA CAT CCG TAA AGA CC-3′. The amplified products were separated by electrophoresis on a 1.5% agarose gel, analyzed using the FluorChem 8000 system(Alpha Innotech, Santa Clara, CA, USA) and the Jagged-1/*β-*actin ratio was calculated.

Real-time qPCR was performed on an ABI7300 sequence detection system (Applied Biosystems). The real-time qPCR reaction was amplified with the following procedure: 95°C for 10 min, and 35 repeats at 95°C for 30 s, 60°C for 30 s and 72°C for 30 s. The 2^−ΔΔCt^ method was performed for the relative quantification of mRNA expression.

### Western Blot

The cells were treated as described in the “Flow cytometry” section. Six hours before the end of the treatment, the cells were stimulated with 50 ng/ml phorbol-12-myristate-13-acetate (PMA) (Sigma-Aldrich, St. Louis, MO, USA) plus 1 μg/ml of ionomycin (Alexis, Lausen, Switzerland) for the below experiments. Meanwhile, 10 μg/ml of GolgiStop (Becton Dickinson, Franklin Lakes, NJ, USA) was added to the cells. The treated cells were lysed with radio-immunoprecipitation assay (RIPA) lysis buffer (BioColors, Shanghai, China) containing phenylmethanesulfonyl fluoride. The proteins were analyzed on SDS-PAGE gels and electrophoretically transferred onto the nitrocullose membranes. The membranes were incubated with rabbit anti-RORγt (Santa Cruz biotechnology, Santa Cruz, CA, USA), rat anti-IL-17A (Cell Signaling, Danvers, MA, USA) or goat anti-IL-17F (R&D Systems, Minneapolis, MN) primary antibody respectively overnight. The membranes were washed three times with TBST, followed by the incubation with the appropriate HRP-conjugated secondary antibody for 1 hour at the room temperature. The specific bands were identified with an Enhanced Chemiluminescence Detection Kit (Amersham Biosciences, Piscataway, NJ, USA). This test was repeated for at least three times.

### ELISA

The cells were treated as described in the “Flow cytometry” section. Six hours before the end of the treatment, the cells were stimulated with 50 ng/ml phorbol-12-myristate-13-acetate (PMA) (Sigma-Aldrich, St. Louis, MO, USA) plus 1 μg/ml of ionomycin (Alexis, Lausen, Switzerland) for the below experiments. Supernatants were harvested from the above treated cells. To detect IL-17A and IL-17F in the cell culture supernatants, we used the mouse IL-17A ELISA Kit (DAKEWE, Shenzhen, China) or IL-17F ELISA kit (BioLegend, San Diego, CA) according to the manufacturer's instructions. Absorbance value was measured at 450 nm on a 680 type microplate reader (BIO-RAD, Berkeley, CA, USA.)

### Immunofluorescence staining

The isolated CD4^+^ T cells were seeded in a 24-well plate at the density of 1.5 × 10^6^ cells/well and treated as described in the “Flow cytometry” section. The *In situ* expressions of RORγt, IL-17A and IL-17F in the treated CD4^+^ T cells were examined via immunofluorescence staining. The treated cells were fixed in cold methanol at 4°C for 10 min. Then, the cells were washed with PBS and blocked with 5% BSA in PBS for 30 min. The blocked cells were incubated with rabbit anti-RORγt (Santa Cruz biotechnology, Santa Cruz, CA, USA), rat anti-IL-17A (Cell Signaling, Danvers, MA, USA) and goat anti-IL-17F (R&D Systems, Minneapolis, MN) antibodies for 3 h at 4°C. Subsequently, the cells were incubated with a donkey anti-goat IgG-PE antibody (Santa Cruz biotechnology, Santa Cruz, CA, USA), a Alexa Fluor 488-conjugated anti-rat IgG antibody, a Alexa Fluor 488-conjugated anti-rabbit IgG antibody or a Alexa Fluor 647-conjugated anti-rabbit IgG antibody (Cell Signaling Technology, Inc.) for 1 h at 37°C. In addition, 6-diamidino-2-phenylindole (DAPI) or propidium iodide (PI) staining was performed for 5 min at the same temperature. The cells were detected using the Leica DMRA2 fluorescence microscope with FW4000 software (Leica, Germany). Positive cells or negative cells under 10 fields of view selected randomly for each group were counted for average percentages.

### Statistical analysis

Student's t-test was performed for the paired data comparison and one way ANOVA was followed for comparing the sets of more than two groups. P value <0.05 was considered statistically significant.

## Author Contributions

F.Y.X. and J.L. conceived and designed the work. Y.W. performed most of the experiments and statistical analysis. S.Q.Y., J.X., J.F.D. and S.Z. assisted to complete the partial tests. F.Y.X. interpreted the experimental data. Y.W. contributed to the manuscript preparation and F.Y.X. drafted the manuscript.

## Figures and Tables

**Table 1:** 84 genes assessed by qPCR array

Symbol	Name	Symbol	Name
Cacybp	Calcyclin binding protein	Ccl1	Chemokine (C-C motif) ligand 1
Ccl2	Chemokine (C-C motif) ligand 2	Ccl20	Chemokine (C-C motif) ligand 20
Ccl22	Chemokine (C-C motif) ligand 22	Ccl7	Chemokine (C-C motif) ligand 7
Cd2	CD2 antigen	Cd247	CD247 antigen
Cd28	CD28 antigen	Cd34	CD34 antigen
Cd3d	CD3 antigen, delta polypeptide	Cd3e	CD3 antigen, epsilon polypeptide
Cd3g	CD3 antigen, gamma polypeptide	Cd4	CD4 antigen
Cd40lg	CD40 ligand	Cd8a	CD8 antigen, alpha chain
Cebpb	CCAAT/enhancer binding protein (C/EBP), beta	Clec7a	C-type lectin domain family 7, member a
Csf2	Colony stimulating factor 2 (granulocyte-macrophage)	Csf3	Colony stimulating factor 3 (granulocyte)
Cx3cl1	Chemokine (C-X3-C motif) ligand 1	Cxcl1	Chemokine (C-X-C motif) ligand 1
Cxcl12	Chemokine (C-X-C motif) ligand 12	Cxcl2	Chemokine (C-X-C motif) ligand 2
Cxcl5	Chemokine (C-X-C motif) ligand 5	Il25	Interleukin 25
S1pr1	Sphingosine-1-phosphate receptor 1	Foxp3	Forkhead box P3
Gata3	GATA binding protein 3	Icam1	Intercellular adhesion molecule 1
Icos	Inducible T-cell co-stimulator	Ifng	Interferon gamma
Il10	Interleukin 10	Il12b	Interleukin 12B
Il12rb1	Interleukin 12 receptor, beta 1	Il12rb2	Interleukin 12 receptor, beta 2
Il13	Interleukin 13	Il15	Interleukin 15
Il17a	Interleukin 17A	Il17c	Interleukin 17C
Il17d	Interleukin 17D	Il17f	Interleukin 17F
Il17rb	Interleukin 17 receptor B	Il17rc	Interleukin 17 receptor C
Il17rd	Interleukin 17 receptor D	Il17re	Interleukin 17 receptor E
Il18	Interleukin 18	Il1b	Interleukin 1 beta
Il2	Interleukin 2	Il21	Interleukin 21
Il22	Interleukin 22	Il23a	Interleukin 23, alpha subunit p19
Il23r	Interleukin 23 receptor	Il27	Interleukin 27
Il3	Interleukin 3	Il4	Interleukin 4
Il5	Interleukin 5	Il6	Interleukin 6
Il6ra	Interleukin 6 receptor, alpha	Il7r	Interleukin 7 receptor
Isg20	Interferon-stimulated protein	Jak1	Janus kinase 1
Jak2	Janus kinase 2	Mmp13	Matrix metallopeptidase 13
Mmp3	Matrix metallopeptidase 3	Mmp9	Matrix metallopeptidase 9
Myd88	Myeloid differentiation primary response gene 88	Nfatc2	Nuclear factor of activated T-cells, cytoplasmic,calcineurin-dependent 2
Nfkb1	Nuclear factor of kappa light polypeptide gene enhancer in B-cells 1, p105	Rorc	RAR-related orphan receptor gamma
Socs1	Suppressor of cytokine signaling 1	Socs3	Suppressor of cytokine signaling 3
Stat3	Signal transducer and activator of transcription 3	Stat4	Signal transducer and activator of transcription4
Stat5a	Signal transducer and activator of transcription 5a	Stat6	Signal transducer and activator of transcription 6
Sykb	Spleen tyrosine kinase	Tbx21	T-box 21
Tgfb1	Transforming growth factor, beta 1	Tirap	Toll-interleukin 1 receptor (TIR) domain-containing adaptor protein
Tlr4	Toll-like receptor 4	Tnf	Tumor necrosis factor
Traf6	Tnf receptor-associated factor 6	Yy1	YY1 transcription factor

## References

[b1] Harrington L. E. *et al.* Interleukin 17-producing CD4+ effector T cells develop via a lineage distinct from the T helper type 1 and 2 lineages. Nat Immunol 6, 1123–1132 (2005). 16200070 10.1038/ni1254

[b2] Park H. *et al.* A distinct lineage of CD4 T cells regulates tissue inflammation by producing interleukin 17. Nat Immunol 6, 1133–1141 (2005). 16200068 10.1038/ni1261 PMC1618871

[b3] Veldhoen M. *et al.* TGFbeta in the context of an inflammatory cytokine milieu supports de novo differentiation of IL-17-producing T cells. Immunity 24, 179–189 (2006). 16473830 10.1016/j.immuni.2006.01.001

[b4] Li M. O. *et al.* Transforming growth factor-beta regulation of immune responses. Annu Rev Immunol 24, 99–146 (2006). 16551245 10.1146/annurev.immunol.24.021605.090737

[b5] Bettelli E. *et al.* Reciprocal developmental pathways for the generation of pathogenic effector TH17 and regulatory T cells. Nature 441, 235–238 (2006). 16648838 10.1038/nature04753

[b6] Ivanov II *et al.* The orphan nuclear receptor RORgammat directs the differentiation program of proinflammatory IL-17+ T helper cells. Cell 126, 1121–1133 (2006). 16990136 10.1016/j.cell.2006.07.035

[b7] Zhou L. *et al.* TGF-beta-induced Foxp3 inhibits T(H)17 cell differentiation by antagonizing RORgammat function. Nature 453, 236–240 (2008). 18368049 10.1038/nature06878 PMC2597437

[b8] Zhou L. A. *et al.* IL-6 programs TH-17 cell differentiation by promoting sequential engagement of the IL-21 and IL-23 pathways. Nat Immunol 8, 967–974 (2007). 17581537 10.1038/ni1488

[b9] Han H. *et al.* Inducible gene knockout of transcription factor recombination signal binding protein-J reveals its essential role in T versus B lineage decision. Int Immunol 14, 637–645 (2002). 12039915 10.1093/intimm/dxf030

[b10] Radtke F. *et al.* Deficient T cell fate specification in mice with an induced inactivation of Notch1. Immunity 10, 547–558 (1999). 10367900 10.1016/s1074-7613(00)80054-0

[b11] Radtke F., Wilson A. & MacDonald H. R. Notch signaling in T- and B-cell development. Curr Opin Immunol 16, 174–179 (2004). 15023410 10.1016/j.coi.2004.01.002

[b12] Tanigaki K. & Honjo T. Regulation of lymphocyte development by Notch signaling. Nat Immunol 8, 451–456 (2007). 17440450 10.1038/ni1453

[b13] Nyfeler Y. *et al.* Jagged1 signals in the postnatal subventricular zone are required for neural stem cell self-renewal. EMBO J 24, 3504–3515 (2005). 16163386 10.1038/sj.emboj.7600816 PMC1276174

[b14] Amsen D. *et al.* Instruction of distinct CD4 T helper cell fates by different notch ligands on antigen-presenting cells. Cell 117, 515–526 (2004). 15137944 10.1016/s0092-8674(04)00451-9

[b15] Mukherjee S. *et al.* Regulation of T cell activation by Notch ligand, DLL4, promotes IL-17 production and Rorc activation. J Immunol 182, 7381–7388 (2009). 19494260 10.4049/jimmunol.0804322 PMC2980695

[b16] Ito T. *et al.* TLR9 regulates the mycobacteria-elicited pulmonary granulomatous immune response in mice through DC-derived Notch ligand delta-like 4. J Clin Invest 119, 33–46 (2009). 19075396 10.1172/JCI35647 PMC2613456

[b17] Jurynczyk M. *et al.* Notch3 inhibition in myelin-reactive T cells down-regulates protein kinase C theta and attenuates experimental autoimmune encephalomyelitis. J Immunol 180, 2634–2640 (2008). 18250475 10.4049/jimmunol.180.4.2634

[b18] You P. *et al.* Jagged-1-HES-1 signaling inhibits the differentiation of TH17 cells via ROR gammat. J Biol Regul Homeost Ag 27, 79–93 (2013). 23489689

[b19] Alam C. *et al.* Inflammatory tendencies and overproduction of IL-17 in the colon of young NOD mice are counteracted with diet change. Diabetes 59, 2237–2246 (2010). 20547977 10.2337/db10-0147 PMC2927946

[b20] Broberg E. K. *et al.* Herpes simplex virus type 1 infection induces upregulation of interleukin-23 (p19) mRNA expression in trigeminal ganglia of BALB/c mice. J Interf Cytok Res 22, 641–651 (2002). 10.1089/10799900260100123 12162874

[b21] Diefenbach A S. H. *et al.* Requirement for type 2 NO synthase for IL-12 signaling in innate immunity. Science 284, 951–955 (1999). 10320373 10.1126/science.284.5416.951

[b22] Yang X. X. O. *et al.* T helper 17 lineage differentiation is programmed by orphan nuclear receptors ROR alpha and ROR gamma. Immunity 28, 29–39 (2008). 18164222 10.1016/j.immuni.2007.11.016 PMC2587175

[b23] Choi J. W. *et al.* Characterization of the transcriptional expression of Notch-1 signaling pathway members, Deltex and HES-1, in developing mouse thymocytes. Dev Comp Immunol 26, 575–588 (2002). 12031417 10.1016/s0145-305x(01)00095-7

[b24] Hoyne G. F. *et al.* Serrate1-induced Notch signalling regulates the decision between immunity and tolerance made by peripheral CD4(+) T cells. Int Immunol 12, 177–185 (2000). 10653853 10.1093/intimm/12.2.177

[b25] Kuroda K. *et al.* Regulation of marginal zone B cell development by MINT, a suppressor of Notch/RBP-J signaling pathway. Immunity 18, 301–312 (2003). 12594956 10.1016/s1074-7613(03)00029-3

[b26] Singh N. *et al.* Expression of notch receptors, notch ligands, and fringe genes in hematopoiesis. Exp Hematol 28, 527–534 (2000). 10812242 10.1016/s0301-472x(00)00146-6

[b27] Lindsell C. E S. C., Boulter J. & Weinmaster G. Jagged: a mammalian ligand that activates Notch1. Cell 80, 909–917 (1995). 7697721 10.1016/0092-8674(95)90294-5

[b28] Tsukumo S. & Yasutomo K. Notch governing mature T cell differentiation. J Immunol 173, 7109–7113 (2004). 15585829 10.4049/jimmunol.173.12.7109

[b29] Maekawa Y. *et al.* Delta1-Notch3 interactions bias the functional differentiation of activated CD4(+) T cells. Immunity 19, 549–559 (2003). 14563319 10.1016/s1074-7613(03)00270-x

[b30] Vigouroux S. *et al.* Induction of antigen-specific regulatory T cells following overexpression of a Notch ligand by human B lymphocytes. J Virol 77, 10872–10880 (2003). 14512537 10.1128/JVI.77.20.10872-10880.2003 PMC224961

[b31] Yvon E. S. *et al.* Overexpression of the Notch ligand, Jagged-1, induces alloantigen-specific human regulatory T cells. Blood 102, 3815–3821 (2003). 12842995 10.1182/blood-2002-12-3826

[b32] Oppmann B. *et al.* Novel p19 protein engages IL-12p40 to form a cytokine, IL-23, with biological activities similar as well as distinct from IL-12. Immunity 13, 715–725 (2000). 11114383 10.1016/s1074-7613(00)00070-4

[b33] Cua D. J. *et al.* Interleukin-23 rather than interleukin-12 is the critical cytokine for autoimmune inflammation of the brain. Nature 421, 744–748 (2003). 12610626 10.1038/nature01355

[b34] McKenzie B. S., Kastelein R. A. & Cua D. J. Understanding the IL-23-IL-17 immune pathway. Trends Immunol 27, 17–23 (2006). 16290228 10.1016/j.it.2005.10.003

[b35] Langrish C. L. *et al.* IL-23 drives a pathogenic T cell population that induces autoimmune inflammation. J Exp Med 201, 233–240 (2005). 15657292 10.1084/jem.20041257 PMC2212798

[b36] Elson C. O. *et al.* Monoclonal anti-interleukin 23 reverses active colitis in a T cell-mediated model in mice. Gastroenterol 132, 2359–2370 (2007). 10.1053/j.gastro.2007.03.104 17570211

[b37] Mangan P. R. *et al.* Transforming growth factor-beta induces development of the T(H)17 lineage. Nature 441, 231–234 (2006). 16648837 10.1038/nature04754

[b38] Aggarwal S. *et al.* Interleukin-23 promotes a distinct CD4 T cell activation state characterized by the production of interleukin-17. J Biol Chem 278, 1910–1914 (2003). 12417590 10.1074/jbc.M207577200

[b39] Parham C C. M. *et al.* A receptor for the heterodimeric cytokine IL-23 is composed of IL-12Rbeta1 and a novel cytokine receptor subunit, IL-23R. J Immunol 168, 5699–5708 (2002). 12023369 10.4049/jimmunol.168.11.5699

[b40] Mathur A. N. *et al.* Stat3 and Stat4 direct development of IL-17-secreting Th cells. J Immunol 178, 4901–4907 (2007). 17404271 10.4049/jimmunol.178.8.4901

[b41] Yang X. O. *et al.* Watowich, and C. Dong. STAT3 regulates cytokine-mediated generation of inflammatory helper T cells. J Biol Chem 282, 9358–9363 (2007). 17277312 10.1074/jbc.C600321200

[b42] Elyaman W. *et al.* Jagged1 and Delta1 differentially regulate the outcome of Experimental Autoimmune Encephalomyelitis. J Immunol 179, 5990–5998 (2007). 17947672 10.4049/jimmunol.179.9.5990

[b43] Keerthivasan S. *et al.* Notch signaling regulates mouse and human Th17 differentiation. J Immunol 187, 692–701 (2011). 21685328 10.4049/jimmunol.1003658 PMC3131467

[b44] Bailis W. *et al.* Notch simultaneously orchestrates multiple helper T cell programs independently of cytokine signals. Immunity 39, 148–159 (2013). 23890069 10.1016/j.immuni.2013.07.006 PMC3762693

